# Analgesic Effect of Extracorporeal Shock-Wave Therapy in Individuals with Lateral Epicondylitis: A Randomized Controlled Trial

**DOI:** 10.3390/jfmk7010029

**Published:** 2022-03-18

**Authors:** Salameh Aldajah, Anas R. Alashram, Giuseppe Annino, Cristian Romagnoli, Elvira Padua

**Affiliations:** 1 Department of Physiotherapy, Isra University, Amman 11622, Jordan; salameh.aldajah@iu.edu.jo; 2 Department of Systems Medicine, University of Rome “Tor Vergata”, 00133 Rome, Italy; g_annino@hotmail.com; 3 School in Science and Culture of Well-Being and Lifestyle, Alma Mater University, 40126 Bologna, Italy; cristian-romagnoli@outlook.it; 4 Department of Human Sciences and Promotion of the Quality of Life, San Raffaele Rome Open University, 00166 Rome, Italy; elvira.padua@uniroma5.it

**Keywords:** lateral epicondylitis, physiotherapy, shock wave, pain, function

## Abstract

This study was conducted to investigate the effect of extracorporeal shock-wave therapy (ESWT) on pain, grip strength, and upper-extremity function in lateral epicondylitis. A sample of 40 patients with LE (21 males) was randomly allocated to either the ESWT experimental (n = 20) or the conventional-physiotherapy control group (n = 20). All patients received five sessions during the treatment program. The outcome measures used were the Visual Analog Scale (VAS), the Taiwan version of the Disabilities of the Arm, Shoulder, and Hand (DASH) questionnaire, and a dynamometer (maximal grip strength). Forty participants completed the study. Participants in both groups improved significantly after treatment in terms of VAS (pain reduced), maximal grip strength, and DASH scores. However, the pain was reduced and upper-extremity function and maximal grip strength were more significantly improved after ESWT in the experimental group. ESWT has a superior effect in reducing pain and improving upper-extremity function and grip strength in people with lateral epicondylitis. It seems that five sessions of ESWT are optimal to produce a significant difference. Further studies are strongly needed to verify our findings.

## 1. Introduction

Lateral epicondylitis is the most frequent cause of elbow pain in adult individuals [1]. It is a degenerative injury that most frequently occurs in the origin of the common extensor tendon and is associated with activities that place extreme repetitive stress on the lateral forearm and elbow musculature [2]. Applying repetitive stress to a tendon leads to the development of cross-linkages and collagen deposition [3]. When the rate or force of stretching exceeds the tolerance of the tendon, it leads to microtears, and the adaptation of the tendon to multiple microtears leads to tendinosis [2]. The main symptom of lateral epicondylitis is pain at the lateral aspect of the elbow, which is exacerbated by the handgrip that reduces the patients’ grip strength and function [4].

Traditional non-operative treatments of lateral epicondylitis include discontinuation of provocative activities, analgesics, conventional-physical-therapy interventions (i.e., physical modalities), bracing, and corticosteroid injection. However, the impacts of these treatments are either inconsistent or only last for a short duration [2]. 

Extracorporeal shock-wave therapy (ESWT) has shown to be an effective treatment modality in patients with rotator-cuff tendonitis, lateral epicondylitis, and subacromial impingement [5]. ESWT serves as an alternative treatment modality in subjects who reject surgical intervention [6]. Many studies have shown the effectiveness of ESWT on pain reduction among people with lateral epicondylitis [7,8,9]. A randomized controlled trial by Yang et al. (2017) found that patients with lateral epicondylitis had better and faster pain reduction and functional improvement following ESWT plus physiotherapy intervention than those who received only physiotherapy intervention [8]. Another randomized controlled trial by Devrimsel et al. (2014) showed that ESWT seems to be more efficient in pain reduction and improvement of functions than laser-therapy intervention [9]. No study has compared the effectiveness of ESWT intervention alone and traditional physiotherapy intervention. Therefore, this study was conducted to evaluate and compare the effectiveness of ESWT and traditional physiotherapy intervention in lateral-epicondylitis treatment.

## 2. Methods

### 2.1. Participants 

The study was designed as a single randomized controlled trial in which a convenience sample of 40 participants with lateral epicondylitis (19 females, 21 males) was selected based on the study’s inclusion and exclusion criteria. The inclusion criteria were participants aged 18 to 80 years old with a confirmed diagnosis of lateral epicondylitis and lateral elbow pain lasting between 6 to 12 months. Participants were excluded if they had cervical problems, elbow deformity, diabetes mellitus, problems in the thyroid gland, malignancy, pregnancy, and corticosteroid injections to the lateral epicondyle within six weeks. Written informed consent was obtained from participants before they were randomly assigned to either the ESWT experimental group (n = 20) or the conventional-physiotherapy control group (n = 20). Participants were randomized using a computer-generator random sequence of numbers. Study eligibility was identified based on inspection of the participant file. An independent collaborator fulfilled the concealed allocation according to the order of appearance. The study was approved by the Isra University ethics committee with a protocol number (17/173) and was registered in ClinicalTrials.gov with an ID (NCT05142852). This study followed CONSORT checklist (see Supplementary Materials).

### 2.2. Intervention 

This study was performed in the Department of Physiotherapy at Isra University, Amman, Jordan. The included participants received either ESWT or conventional therapy on consecutive days by the same therapist. The participants in the experimental group underwent 5 sessions of ESWT. ESWT was set at 2000 shock waves with 1.6 bar intensity and 16 Hz frequency using the Swiss DolorClast Master (EMS, Nyon, Switzerland) [9]. The control group underwent 5 sessions of conventional-physical-therapy (CPT) intervention for 5 min. The control intervention consisted of a 1 min friction massage, followed by 3 min of continuous therapeutic ultrasound with a frequency of 1.5 Hz and a 1 min direct-ice massage over the elbow common-extensor tendon. All participants used 10 cm lateral epicondyle bandages in the treatment period.

### 2.3. Outcome Measures

The participants in both groups were clinically assessed at baseline (before the first session) and the end of the treatment sessions (end of the fifth session) by one assessor blinded to the intervention. Demographic information from each participant, including age, sex, history of upper-limb injuries, and history of chronic diseases, was collected. The pain intensity, maximal grip strength, upper-extremity disability and symptoms for each participant were assessed. The assessor was blinded to the intervention.

#### 2.3.1. Primary Outcome Measure

##### Pain Intensity

Participants were asked to rate their present pain intensity, as caused by the lateral epicondylitis, from 0 to 10 using the Visual Analog Scale (VAS). If participants had bilateral lateral epicondylitis, the side with the worse pain intensity was chosen for the evaluation.

#### 2.3.2. Secondary Outcome Measure

##### Upper-Extremity Function 

Upper-extremity disability was assessed using the Taiwan version of the Disabilities of the Arm, Shoulder, and Hand (DASH) questionnaire [10]. This questionnaire consists of 21 different tasks on a 5-point scale. The summary score was transformed to a score out of 100, with a lower score indicating less disability. The internal consistency was excellent, with a Cronbach alpha of 0.96 for the disability/symptom scale and work module and 0.97 for the sports/music module [10]. 

##### Grip Strength 

The maximal grip strength of the affected arm was assessed using a grip-strength dynamometer (Exacta Hydraulic Hand Dynamometer, North Coast Medical Inc., Gilroy, CA, USA). Patients were asked to grip the dynamometer as hard as possible 3 times at 10 s rest intervals, with 90 degrees of elbow flexion, shoulder adduction, slight extension in the wrist, and the forearm in the neutral position [11]. The highest grip-strength number was registered. 

### 2.4. Statistical Analysis 

The Shapiro–Wilk test was used to check the data normality. At baseline, the participants’ characteristics in each group were analyzed using frequencies and descriptive analysis. The Mann–Whitney U test for 2 independent samples was used to evaluate the mean difference in the outcome measures between the groups at the end of the treatment program. A nonparametric measure for the related sample (Wilcoxon Signed-Ranks test) was used to evaluate the mean difference within each group in the period between baseline and end of the treatment program. The significant difference was set at *p* < 0.05. Effect sizes (ES) were calculated to identify the difference between the baseline and post-test values of the same group using the following formula: ES = Z/√N (Z: Z value, N: number of observations). The small effect size was set at 0.1, moderate at 0.3, and large at 0.5 [12]. The effect size between groups was identified using Cohen’s *d* by dividing the difference between the means of the experimental and control groups by the pooled standard deviation [13]. A value greater than 0.8 was considered large, 0.5 was moderate, and less than 0.2 was small [14]. The sample-size calculation was not performed. Statistical analysis was performed using SPSS statistics version 22.

## 3. Results 

**Figure 1:**
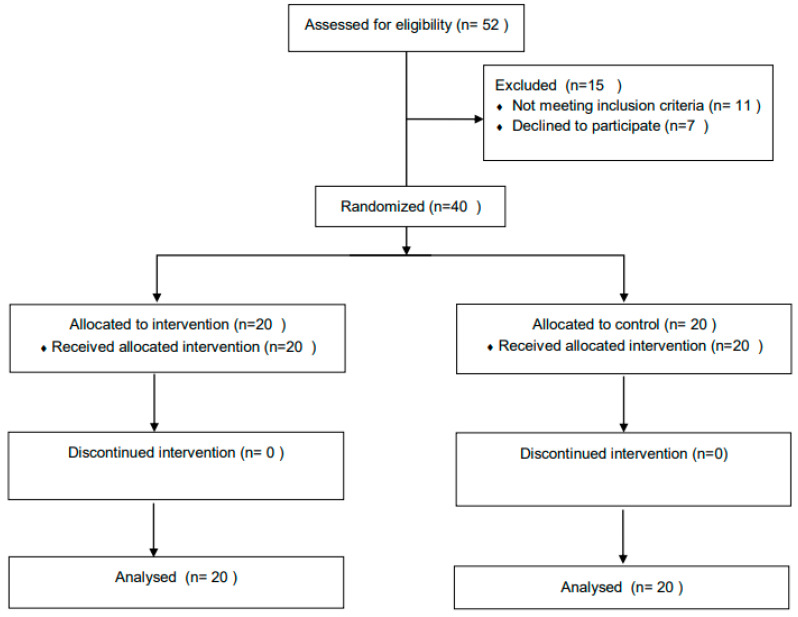
Flow of participants through trial.

Forty participants (20 in the ESWT group and 20 in the conventional-physical-therapy group) completed the study (Figure 1). Demographic and health-related characteristics of the participants are presented in Table 1. No significant differences were found between the two groups, neither in demographic information nor in outcome measures at baseline.

Table 2 shows the means and standard deviations of outcome-measure scores in the treatment (ESWT) and control (conventional physical therapy) groups at baseline and at the end of treatment. Participants in both groups improved significantly after treatment in VAS, MGS and DASH; however, the VAS, MGS, and DASH scores were more significant after the experimental intervention (*p* < 0.000). After investigating the differences between the groups at the end of the treatment, the participants in the ESWT experimental group performed better than those in the conventional-physical-therapy control group in the VAS, DASH, and MGS (*p* < 0.000).

## 4. Discussion 

In this study, we investigated the effects of extracorporeal shock-wave therapy on elbow pain, upper-extremity function, and maximal grip strength in participants with lateral epicondylitis. Similar to the results of our study, Devrimsel et al. (2014) showed that ESWT is more efficient in reducing pain and improving arm function and grip strength in participants with lateral epicondylitis than laser therapy [9]. Another study by Yang et al. (2017) found that participants with lateral epicondylitis had better and faster pain reduction and improvement in upper-extremity function and grip strength after receiving ESWT plus physical-therapy intervention than those who received only physical therapy [8]. 

ESWT activates angiogenesis and promotes blood supply through the tendon-bone area by a rise in angiogenic growth factors in the Achilles’ tendons; accordingly, inflamed tissues are regenerated, and the pain is relieved [15,16]. A study found that ESWT was an effective treatment option in calcific tendinitis of the rotator cuff and chronic plantar fasciitis [17]. In the study by Chen et al. (2004), ESWT was reported to be a suitable modality in Achilles’ tendinitis [18]. ESWT was also shown to be effective in reducing pain in chronic lateral epicondylitis [19,20]. 

ESWT consumes energy at the interface of two substances with varying acoustic impedance, such as the bone-tendon interface, resulting in the release of kinetic energy at the junctions that can induce tissue alterations [21]. It has been proven that ESWT works by exciting nerve fibers to produce analgesia and that disruption of the tendon tissue may induce a healing process [22,23].

Pain reduction and improvement in upper-extremity function are the main goals of lateral-epicondylitis treatment [9]. Lateral epicondylitis has a 1–3% prevalence in the general population, while this percentage increases in individuals aged between 30–60 [24]. The dominant hand is generally more frequently affected, which is explained by the role of physical stress in the pathogenesis of lateral epicondylitis [24]. In the present study, lateral epicondylitis was more frequent in the dominant side of the participants.

In the study by Devrimsel et al. (2014), the participants in the experimental group received ESWT for 10 sessions, with 2000 impulses per session and 16 Hz frequency [9]. Further, in 2017, Yang et al. demonstrated significant improvement in VAS (pain reduced), maximal grip strength, and DASH scores immediately following 5 min of ESWT combined with conventional-physiotherapy intervention [8]. In their study, each participant in the experimental group received ESWT for three sessions. Each session consisted of 2000 impulses per session, once a week over 3 weeks (a total of 6000 shock waves) with a frequency set at 10 Hz, followed by a low-frequency electric therapy apparatus, ultrasound diathermy, and 10 min of static stretching exercise plus a transverse friction massage of the affected elbow over the common-extensor tendon. In the current study, we used the same number of impulses for each treatment session (2000 impulses) with a frequency of 16 Hz, 5 min ESWT for five consecutive sessions.

In the current study, the participants in the control group showed a significant reduction in pain and improvement in the upper-extremity function after a 5 min CPT intervention. Although our findings are consistent with several studies that administered the CPT interventions for 10 to 15 min, administrating CPT for 10 to 15 min may produce different results compared with 5 min CPT. A study by D’vaz et al. (2005) showed a significant reduction in pain and improvement in the upper-extremity function and grip strength after ultrasound intervention [25]. Another study showed that friction massage combined with therapeutic ultrasound reduces pain in individuals with lateral epicondylitis [26]. Moreover, a study showed that cryotherapy is effective in reducing pain in patients with lateral epicondylitis [27].

Therapeutic ultrasound is a widely used practice in physiotherapy and sports medicine to treat different injures, and this method is focused on changing the extensibility of the collagenous tissues to improve the range of motion [28]. It has been shown that therapeutic ultrasound reduces pain and improves upper-extremity function and grip strength in participants with lateral epicondylitis [29]. The physiological influences of therapeutic ultrasound include increased tissue temperature [30], improved local blood flow [31], increased extensibility of tissue [32], and reduced viscosity of fluid elements in the body tissue [33]. Additionally, the mechanical effects accelerate tissue metabolism by enhancing cellular permeability and ion transport [34].

On the other hand, deep friction massage affects muscle tissue in the vertical direction of fibers [35]. Mechanically, deep friction massages cause hyperemia, rearrange collagen in normal soft tissue, reduce inflammation, and decrease pain through “barrier regulation theory”. It has been demonstrated that it destroys or prevents abnormal fiber adhesions, decreases stress, remodels collagen, and enhances the quality of wound tissue [36]. Recently, Lee et al. (2020) reported that combined deep friction massage and taping intervention can be a more effective treatment strategy for decreasing pain, improving upper-extremity function in participants with lateral epicondylitis than intervention by taping alone [37]. Finally, Whaley and Baker (2004) recommended using ice three times per day for 15 min to reduce the inflammation by decreasing the level of chemical activity and vasoconstriction, which reduces the swelling [38].

Many interventions have proven their effectiveness in reducing pain and improving upper extremity in participants with lateral epicondylitis, such as stretching, theraband exercises, flexbar exercises, and taping [39,40]. Devrimsel et al. (2014) demonstrated an improvement in upper-extremity function and grip strength, as well as a reduction in pain [9]. Accordingly, we propose that adding ESWT to conventional-physiotherapy intervention may show a superior effect in lateral-epicondylitis recovery than intervention by ESWT alone.

This study has many limitations that should be acknowledged. Firstly, this study is not a double-blinded study. However, using an instrumented measure for outcome assessment and blinded, independent assessors of the groups partially limited this bias. Secondly, the present study included a small number of patients. Future studies should have a larger sample size to prove our results. Lastly, the participants with lateral epicondylitis were tested only before and after the intervention without a long follow-up. To understand the effect of ESWT in participants with lateral epicondylitis, future studies should include a long follow-up.

## 5. Conclusions 

Our data suggest that five sessions of ESWT intervention showed a significant reduction of pain and determined significant improvement in upper-extremity function and grip strength. Further studies are warranted to verify our findings.

## Figures and Tables

**Table 1:** Characteristics of participants.

Characteristic	Shock-Wave Experimental Group (n = 20)	CPT Control Group (n = 20)	All Participants (n = 40)	*p* Value
Age: mean ± SD	42.0 ± 7.30	42.37 ± 6.69	42.28 ± 6.91	0.923
Gender: n (%)				0.513
Male	11	10	24	
Female	9	10	19	
Weight	81.10 ± 13.30	81.89 ± 11.65	81.49 ± 12.37	0.967
High	1.77 ± 0.14	1.77 ± 0.15	1.77 ± 0.14	0.989
Injury duration (Months)	8.41 ± 1.33	9.23 ± 1.17	8.82 ± 1.25	0.671
Right/Left elbow	17/5	20/1	37/6	0.678

CPT: Conventional Physiotherapy.

**Table 2:** Mean and standard deviation (SD) of outcome measures for Shock-wave experimental group and conventional-physical-therapy (CPT) control group at baseline and post-treatment (N = 40).

	Within Group Difference								Between Groups Difference	
	Shock-Wave Experimental Group (n = 20)				CPT Control Group (n = 20)					
	Baseline Mean ± SD	Post-treatment Mean ± SD	Effect size	*p*-Value	Baseline Mean ± SD	Post-treatment Mean ± SD	Effect size	*p*-Value	Cohen’s *d*	*p*-Value ^$^
VAS	8.25 ± 0.72	1.75 ± 0.85	0.18	0.000 *	8.11 ± 0.81	3.37 ± 0.83	0.17	0.008 **	0.93	0.000 *
DASH	59.70 ± 5.50	48.89 ± 2.77	0.17	0.000 *	57.94 ± 6.15	54.47 ± 6.44	0.14	0.005 **	0.83	0.000 *
MGS	23.20 ± 3.81	27.48 ± 2.69	0.21	0.000 *	24.95 ± 3.96	25.68 ± 3.51	0.19	0.033 **	0.81	0.029 **

VAS: Visual Analogue Scale; DASH: Disabilities of the Arm, Shoulder and Hand; MGS: Maximal grip strength, * *p* < 0.001, ** *p* < 0.05, ^$^: Between groups difference.

## Supplementary Materials

The following supporting information can be downloaded at: https://www.mdpi.com/article/10.3390/jfmk7010029/s1, This study followed CONSORT.
